# Translation of evidence into kidney transplant clinical practice: managing drug-lab interactions by a context-aware clinical decision support system

**DOI:** 10.1186/s12911-020-01196-w

**Published:** 2020-08-20

**Authors:** Zahra Niazkhani, Mahsa Fereidoni, Parviz Rashidi Khazaee, Afshin Shiva, Khadijeh Makhdoomi, Andrew Georgiou, Habibollah Pirnejad

**Affiliations:** 1 Nephrology and Kidney Transplant Research Center, Urmia University of Medical Sciences, Urmia, Iran; 2 Department of Health Information Technology, Urmia University of Medical Sciences, Urmia, Iran; 3 Student Research Committee, Urmia University of Medical Sciences, Urmia, Iran; 4 Faculty of IT and Computer Engineering, Urmia University of Technology, Urmia, Iran; 5 Department of Clinical Pharmacy, Urmia University of Medical Sciences, Urmia, Iran; 6 Department of Adult Nephrology, Urmia University of Medical Sciences, Urmia, Iran; 7 Centre for Health Systems and Safety Research, Australian Institute of Health Innovation, Macquarie University, Sydney, Australia; 8 Patient Safety Research Center, Urmia University of Medical Sciences, Urmia, Iran; 9 Erasmus School of Health Policy & Management (ESHPM), Erasmus University Rotterdam, Rotterdam, the Netherlands

**Keywords:** Drug-lab interactions, Clinical decision support systems, Medication errors, Kidney transplant, Patient safety

## Abstract

**Background:**

Drug-laboratory (lab) interactions (DLIs) are a common source of preventable medication errors. Clinical decision support systems (CDSSs) are promising tools to decrease such errors by improving prescription quality in terms of lab values. However, alert fatigue counteracts their impact. We aimed to develop a novel user-friendly, evidence-based, clinical context-aware CDSS to alert nephrologists about DLIs clinically important lab values in prescriptions of kidney recipients**.**

**Methods:**

For the most frequently prescribed medications identified by a prospective cross-sectional study in a kidney transplant clinic, DLI-rules were extracted using main pharmacology references and clinical inputs from clinicians. A CDSS was then developed linking a computerized prescription system and lab records. The system performance was tested using data of both fictitious and real patients. The “Questionnaire for User Interface Satisfaction” was used to measure user satisfaction of the human-computer interface.

**Results:**

Among 27 study medications, 17 needed adjustments regarding renal function, 15 required considerations based on hepatic function, 8 had drug-pregnancy interactions, and 13 required baselines or follow-up lab monitoring. Using IF & THEN rules and the contents of associated alert, a DLI-alerting CDSS was designed. To avoid alert fatigue, the alert appearance was considered as interruptive only when medications with serious risks were contraindicated or needed to be discontinued or adjusted. Other alerts appeared in a non-interruptive mode with visual clues on the prescription window for easy, intuitive notice. When the system was used for real 100 patients, it correctly detected 260 DLIs and displayed 249 monitoring, seven hepatic, four pregnancy, and none renal alerts. The system delivered patient-specific recommendations based on individual lab values in real-time. Clinicians were highly satisfied with the usability of the system.

**Conclusions:**

To our knowledge, this is the first study of a comprehensive DLI-CDSS for kidney transplant care. By alerting on considerations in renal and hepatic dysfunctions, maternal and fetal toxicity, or required lab monitoring, this system can potentially improve medication safety in kidney recipients. Our experience provides a strong foundation for designing specialized systems to promote individualized transplant follow-up care.

Contributions to the literature

- Although, drug laboratory interactions are a common source of preventable medication errors, sustainable interventions to recognize and manage these interactions have not widely been implemented.
- This study reports the processes through which a multidisciplinary team developed a user-friendly, context-aware computerized Clinical Decision Support System (CDSS) to support clinicians in the safe medication prescribing alongside individual patients’ laboratory values.
- When applied, the system delivered patient-specific recommendations on the basis of individual lab values of kidney recipients in real-time.
- Applying patient- and clinical context-specific warnings and reminders on dosing recommendations and conflicts with patients’ lab based safety considerations, especially in high risk patient populations such as organ transplant recipients, will make clinicians to embrace such systems readily, leading to promotion of patient safety.

## Background

Lack of effective laboratory-pharmacy linkage results in drug errors commonly called drug-lab interactions (DLIs). Schiff et al. in their seminal review defined these interactions as errors related to drug choice, drug dosing, laboratory monitoring, lab result interpretation, and broader quality improvement [1]. DLIs are very common in medical practice, especially because new drugs are increasingly discovered and used for patients causing poly-pharmacy. For example, regarding laboratory monitoring, a systematic review highlighted that inadequate monitoring and/or ignoring lab results when prescribing a medication is one of the most frequent errors of *omission* associated with preventable adverse drug events (pADE) and related hospital admissions in ambulatory care [2]. In a multicenter study, it became evident that 39% of initial drug dispensing occurred without the recommended laboratory monitoring [3]. This type of error happened at 23–58% of older people receiving cardiovascular medications [4]. For a drug causing potential life-threatening liver failure, such as Troglitazone, the compliance with recommended liver function laboratory monitoring was as low as 5% despite four consecutive food and drug administration warnings [5]. In spite of extensive recommendations to prevent DLIs, low compliance is a major source of pADE and warrants heightened attention [6].

Due to the multiplicity of DLIs per individual drugs, unaided physicians are unable to remember all DLIs and track them while prescribing [1]. This was shown when the performance of a computer program on detecting such interactions was compared with that of clinicians [7]. Therefore, without systematic efforts to translate the emerging evidence into clinical practice, the existing gap between these two would be continued, and even deepened [8]. Clinical decision support systems (CDSSs) are promising tools to improve compliance with evidence-based recommendations and to avoid such omissions in the practice [9]. Studies have shown that CDSSs have improved initial and follow up laboratory monitoring after a drug therapy [3, 10, 11], guided physicians when excessive doses of drugs need adjustment regarding renal or hepatic function [12–14], and improved ordering behavior in terms of DLI recommendations [15–17]. However, not all CDSSs are smoothly embraced by physicians and alert fatigue is a serious concern counteracting their impact. Systematic reviews found that such interventions succeed more commonly when the decision support is provided to clinicians automatically as a part of the clinicians’ workflow, considered as an integrated component of charting or order entry systems and contain recommendations rather than notifications of the patient condition [18, 19]. Regarding alert presentation, studies have shown that clinicians prefer concise, selective, specialty- and patient-specific alerts, which are delivered in an active, more intrusive mode for alerts with high critical importance (e.g., necessity to acknowledge pop-up interruptions for drug contraindications) [20, 21].

Kidney recipients have fragile kidney and liver organ functions because of their new kidney and the use of drugs that are cleared from the body through the kidney or liver. Drugs with renal clearance, for instance, can cause overdose and toxicity in the case there is a decline in the function of transplanted kidneys. Therefore, the value of detecting DLIs in these patients becomes especially important. Kidney recipients also have poly-pharmacy, with frequent use of complex therapeutic transplant medication regimens, some with a narrow therapeutic index potentially causing nephrotoxicity, hepatotoxicity, electrolyte or metabolic imbalances. They have also some accompanying comorbidities or frequent opportunistic infections, which demand additional medication therapy. All these complexities make managing different kinds of drug interactions a difficult task for unaided physicians while prescribing. Although computerized systems have long been introduced to organ transplantation care [22, 23], the routine application of these systems has yet to be commonplace [24]. As such, there are very few reports of using computerized systems to manage medications in conjunction with laboratory values in transplantation [25–27]. Notably, these systems mainly focused on monitoring blood trough levels of immune-suppressive medications [25, 26]. To our knowledge, studies of computerized systems being used to manage DLIs are missing in the transplantation field. As part of a longitudinal project to improve the quality of kidney transplantation care (see for example [28, 29]), we examined the existing complexities in the context of transplantation care, on the one hand, and the current evidence on DLIs, on the other hand, to develop an evidence-based computerized CDSS to support nephrologists in the safe medication prescribing alongside individual patients’ laboratory values. The processes of this CDSS’s development can be used as a proof-of-concept helping other transplant programs to design and apply more specialized, transplant focused CDSSs.

## Methods

### Study setting

The outpatient transplant clinic of the Urmia Medical Science University (UMSU) has recently implemented a homegrown, renal transplant management system (RTMS). This system has been equipped with a computerized provider order entry (CPOE), a drug-drug interaction CDSS [28] and a glomerular filtration rate prediction CDSS for upcoming follow up visits [29, 30]. Having such a system available in our clinic provided a unique opportunity to develop a DLI-CDSS, alongside others. The implementation of the RTMS was started in a stepwise fashion, primarily involving non-physician care providers in the pre-transplant through post-transplant care processes such as inpatient and outpatient transplant nurses and transplant operating room staff in fall 2018. At the time of this report, we are planning to implement the CPOE and CDSS modules of the RTMS for our nephrologists. The research ethics committee of the UMSU reviewed and approved this project.

### Knowledgebase for the CDSS

#### Selection of medications and the development of algorithms

In this study, the main focus was on the common medications used orally amongst adult outpatients, and not on those largely used in the inpatient setting or via parenteral routes. These medications were previously identified through a prospective cross-sectional study in the clinic during 2 months [28]. Then, the DLIs for the oral administration dosages of these medications were extracted from the main pharmacology textbooks and references, including the “Mosby’s diagnostic and laboratory test reference” [31], the “up-to-date” [32] as well as the “prescriber’s digital reference” [33]. The extracted information was then turned into IF and THEN rules (e.g., IF hepatic impairment exists, THEN use Furosemide with caution) and compiled in a knowledgebase.

DLIs have been categorized as errors related to drug choice (e.g., lab-based indications and contraindications), drug dosing (e.g., renal, hepatic, blood level- guided adjustments), laboratory monitoring (e.g., lab signals of toxicity, baseline and ongoing monitoring), lab result interpretation (e.g., drug interfering with a lab test), and broader quality improvement (e.g., surveillance for unrecognized toxicity and monitoring clinician response delays) [1]. Our decision support rules were in line with these DLI categories, in terms of the adequate responses (indication/contraindication/dose adjustment) to laboratory evidence of renal and/or hepatic inflammation or toxicity, medication-pregnancy contraindications, and indications to monitor relevant lab tests. IF and THEN rules were written in a way to discriminate medications, gender, and abnormal laboratory threshold levels. In addition to the literature, we consulted with clinicians to set the laboratory cutoff values for triggering a rule according to the transplant care context. The appropriate algorithms were then developed to facilitate their incorporation into the knowledge base. These algorithms were reviewed by one clinical pharmacologist and then by the head of our nephrology department who is a senior nephrologist with more than 30 years of clinical experience. Modifications were made to address the clinical context in which transplant follow-up care was delivered. Verification by our clinicians helped us to determine top priorities from a clinical point of view and put aside trivial DLI-considerations. On this basis, we excluded rules for the monitoring of routine lab tests that existed in the kidney transplant follow-up protocols, including complete blood counts, lipid profiles, renal, and liver function tests, and immunosuppressive blood trough levels. These exclusions aimed to mitigate the number of monitoring alerts that would most probably be ignored or inactivated later on.

For estimating renal function, creatinine clearance (CrCl) was automatically calculated using the Cockcroft and Gault formula [34]. For calculation of the lean body weight/mass (LBM), the Hume formula was used [35] [Additional file 1]. We triangulated the detection of liver dysfunction by combining different liver function tests, the history of cirrhosis, and the variables used to calculate clinical scores such as the Child-Pugh score.

A time frame was set to fire the alerts, wherever prior test values were necessary for triggering a rule. The upper threshold for this time frame was set to 6 months in which the system uses historical laboratory values.

#### Development of a workflow based CDSS

The content and the layout of alerts and their appearance were decided after multiple meetings among this project’s team members including a nephrologist and a clinical pharmacologist. After evaluating different scenarios, a consensus was reached on a final alert display. Unified Modeling Language (UML) was used to draw workflow diagrams embedded in the CDSS. After incorporating the final criteria lists into the rules, they were used to program the CDSS system using the C#.Net programming language and SQL Server databases. The designed DLI-CDSS was integrated into the CPOE component of the RTMS.

### A pilot test of the CDSS

To test the DLI-CDSS, we defined 250 fictitious patients to test different algorithms associated with individual drugs, according to varying patient demographics (e.g., gender), clinical conditions (e.g., LBW and CrCl), and lab values (Cr and LFT). Additionally, we pilot tested the system with 100 real patients’ data prospectively collected at our clinic in September 2018. The aim was to achieve the optimal sensitivity, specificity, and accuracy of this DLI-CDSS in detecting DLIs according to our extracted rules. This verification was done by manual review and comparison of these patients’ profiles and the system’s performance.

### Evaluation of user satisfaction of the human-computer interface

We used the “Questionnaire for User Interface Satisfaction (QUIS)” [36] to evaluate clinician-user satisfaction of the system. This tool assesses users’ subjective satisfaction with specific aspects of the human-computer interface and is one of Health Information Technology (HIT) usability study methods [37]. It has six questions for an overall measure of satisfaction and 21 main component questions to measure user satisfaction in four specific interface aspects (i.e., screen factors, terminology and system feedback, learning factors, and system capabilities). Two nephrologists and three clinical pharmacologists participated in this phase. They first attended a quick, direct training session on system use and then worked with it using different patient scenarios. Finally, they were asked to rate their satisfaction with the system on a five-point Likert scale. A mean of three and above was considered a higher satisfaction with the system.

## Results

### Selected medications for the knowledge base

**Fig. 1:**
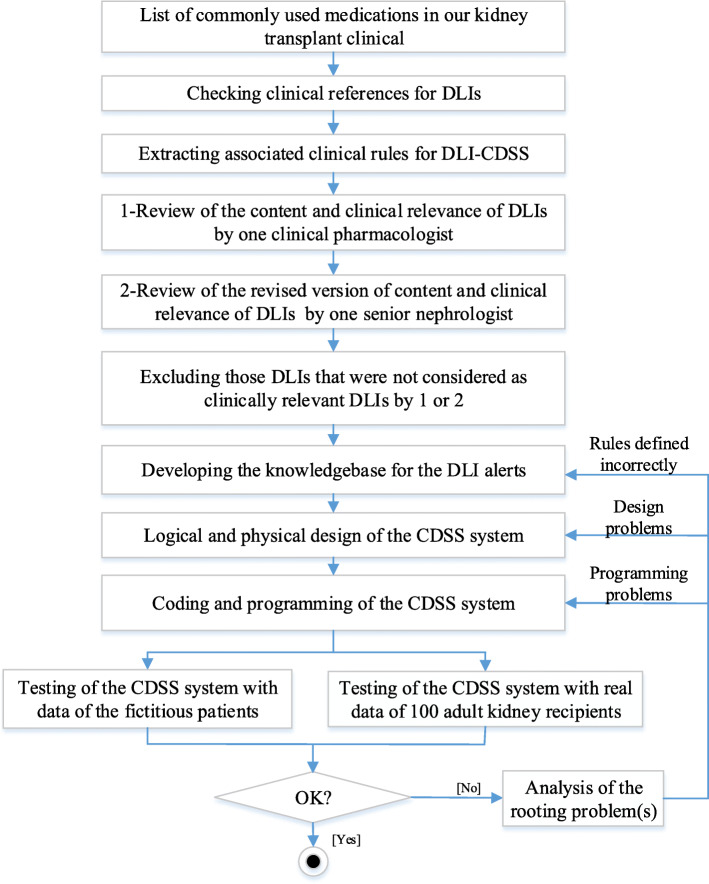
Study flow for designing the DLI-CDSS

For 595 kidney recipients, 29 immunosuppressive and non-immunosuppressive medications constituted 83% of all prescribed drugs. Based on our references and expert opinion, Prazosin and Prednisolone did not have high priority DLI-considerations, which meant that 27 medications were selected [Additional file 2]. Table 1 presents a subset of these selected medications. Figure 1 depicts the process flow in our study.

**Table 1:** Drug-lab interaction guidelines for a sample of our selected high-volume medications

	Medication ^a^	ATC category and name ^b^	Dose adjustment in renal impairment	Dose adjustment in hepatic impairment	Pregnancy considerations	Lab monitoring considerations
1	Allopurinol	M04: ANTIGOUT PREPARATIONS	• 10 ≤ CrCl < 20 ml/min/1.73m^2^: prescribe 200 mg/day per oral • 3 ≤ CrCl < 10 ml/min/1.73m^2^: prescribe 100 mg/day per oral • CrCl < 3 ml/min/1.73m^2^: prescribe 100 mg per oral every 24 h or longer; or 100 mg per oral every third day.	Dosage adjustment may be necessary; No specific recommendations available	–	Monitor Uric acid level: • If normal, check it every 6 months. • If abnormal, change Allopurinol dose accordingly
2	Azathioprine	L04: IMMUNOSUPPRESSANTS	• If renal impairment or oliguria exists, then dosage should be modified depending on clinical response and degree of renal impairment. No quantitative recommendations are available.	Specific guidelines for dosage adjustments in hepatic impairment are not available	Discontinue	–
3	Captopril	C09: AGENTS ACTING ON THE RENIN-ANGIOTENSIN SYSTEM	• 10 ≤ CrCl < 50 ml/min/1.73 m^2^: reduce the recommended dose by 25%. • CrCl < 10 ml/min/1.73 m^2^: reduce the recommended dose by 50%.	No adjustment is required	Discontinue	Monitor Na and K at the baseline and 1–2 weeks after the start.
4	Cyclosporine	L04: IMMUNOSUPPRESSANTS	No adjustment is required	• In hepatic impairment (ALT > 40 U/mL OR AST > 40 U/L OR Bili-total > 1.5 mg/dl): monitor Cyclosporine blood concentration level. May require dose reduction based on concentration.	–	Monitor Uric acid, K, and Mg levels every 2 weeks in the first 3 months then monthly
5	Hydrochlorothiazide	C03: DIURETICS	• CrCl < 30 ml/min/1.73 m^2^: do not use.	• In hepatic impairment (ALT > 40 U/mL OR AST > 40 U/L OR Bili-total > 1.5 mg/dl): use with caution, since minor alteration of fluid and electrolyte balance may precipitate hepatic coma.	–	Monitor K, Na and Cl
6	Losartan	C09: AGENTS ACTING ON THE RENIN-ANGIOTENSIN SYSTEM	• CrCl < 30 ml /min/1.73m^2^: If the patient is also volume-depleted, dose adjustment will be needed.	• In hepatic impairment (ALT > 40 U/mL OR AST > 40 U/L OR Bili-total > 1.5 mg/d): initiate with 25 mg per oral once daily.	Discontinue	Monitor Na and K at the baseline and 1–2 weeks after the start
7	Mycophenolat (mycophenolic acid)	L04: IMMUNOSUPPRESSANTS	• CrCl < 25 ml/min/1.73 m^2^: do not exceed 1 g per oral twice daily.	No adjustment is required	Discontinue	Monitor pregnancy test
8	Omeprazole	A02: DRUGS FOR ACID RELATED DISORDERS	No adjustment is required	• In severe hepatic disease (AST > 120 U/L OR ALT > 120 U/ml OR Bili-total > 3 mg/d) and cirrhotic liver disease: reduce Omeprazole dose to 10 mg once daily receiving for long-term therapy.	–	Monitor Mg and Vit B12 levels periodically

Abbreviations: *ATC-code* Anatomical Therapeutic Chemical-code, *CrCl* Creatinine clearance, *AST* Aspartate aminotransferase, *ALT* Alanine aminotransferase, *Bili* Bilirubin, *Mg* Magnesium, *P* Phosphorus, *K* Potassium

^a^Medications are ordered alphabetically

^b^Drug categories are according to the WHO’s ATC standard

### Knowledgebase for the DLI-CDSS

**Fig. 2:**
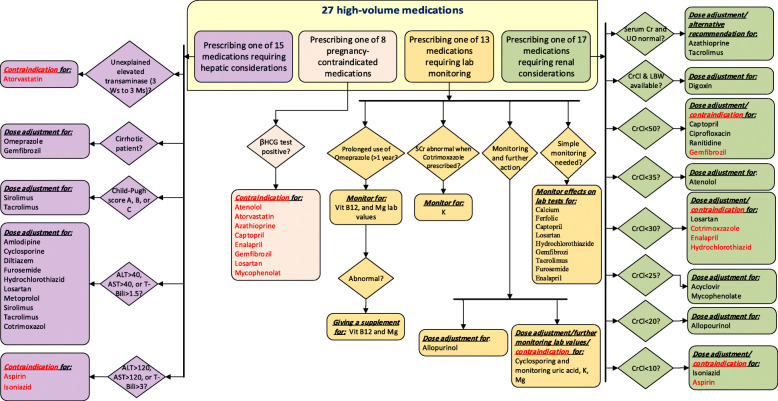
A summary decision rules embedded in the DLI-CDSS

Among 27 medications, 17 medications needed adjustments regarding renal function, 15 required considerations based on hepatic function, eight medications had drug-pregnancy interaction, and 13 required baselines or follow-up lab monitoring (Table 1). Figure 2 shows a general algorithm based upon DLIs dealt with in this study. It simplifies the decision and screening process according to the individual patients’ high-risk condition. A sample of diagrams for individual drugs is provided in the additional materials [see Additional files 3, 4, 5, 6].

The knowledgebase included the IF & THEN rules and contents associated with the alerts. The contents fell under three main categories of “drug selection (indication and contraindication)”, “dosing”, and “lab monitoring”.

#### Algorithms for DLIs in renal impairment

The renal function of individual patients based on CrCl was used to trigger alerts for 17 medications, prompting physicians to either adapt the dose, to choose an alternative drug, or to avoid it. Alert contents for dose adaptation involved: indicating an exact dose (e.g., prescribe 100 mg/day), suggesting a dose reduction (e.g., reduce the recommended dose by 50%), suggesting an extension in the dosing intervals (e.g., extend the dose interval to 12 h), or giving a limit for a maximum daily dose allowed (e.g., do not exceed 25 mg/day). For digoxin, the suggestions for an exact dose were based on individual patients’ CrCl and LBM.

#### Algorithms for DLIs in hepatic impairment

There were 15 medications needing an alert based on hepatic function. The alerts provided recommendations on dose initiation (e.g., initiate with 2.5 mg/day), dose reduction (e.g., reduce dose by 50%), taking caution (e.g., use with caution, since minor alterations of fluid and electrolyte balance may precipitate hepatic coma), or avoiding a drug.

#### Algorithms for drug-pregnancy interaction

In the case of pregnancy, warnings were provided to discontinue eight medications. For mycophenolate, there was also another type of alert that recommended monitoring the status of pregnancy through periodic βHCG tests (i.e., 8 to 10 days after the initiation and then every 3 months afterward). These algorithms were enhanced by a simple rule to have the alerts expire after 9 months or whenever a pregnancy termination was recorded in the RTMS.

#### Algorithms for monitoring drug-lab tests

In this category, alerts prompted physicians to request lab tests for monitoring at the baseline and/or during follow-ups. Additionally, in the case when an abnormal lab value was entered into the system for a monitored lab value, there were alerts to recommend on either dose adjustments, the prescription of a new supplementary medication (e.g., vitamin B12 or magnesium) or avoiding the medication (Fig. 2).

### Alert workflow

#### Alert priority level in the workflow

Whenever the medication data and/or lab values of a patient were changed in the RTMS, the rule-based decision support module of the system matched the changes to the knowledge base in real-time and the associated algorithms were triggered to present patient-specific recommendations to the nephrologist. We placed each alert into one of three levels of clinical severity: level 1 alerts for a fatal or life-threatening interaction, level 2 alerts for an undesirable interaction with the potential for serious injury, and level 3 alerts for required monitoring. For easier visual comprehension, they were displayed by the color codes of red, orange, and yellow, respectively.

#### Alerts’ display rules

While the rules were processed in the background, the display of the alerts was interruptive (i.e., appeared as a pop-up) only when a rule was either contraindicated by a medication’s initiation/ continuation, or when it recommended adjusting the dose (Table 1). While using this system, physicians were not obliged to respond to the alert, or adhere to it; they were free to either make changes to the prescription or continue to proceed with their prescription plan.

**Fig. 3:**
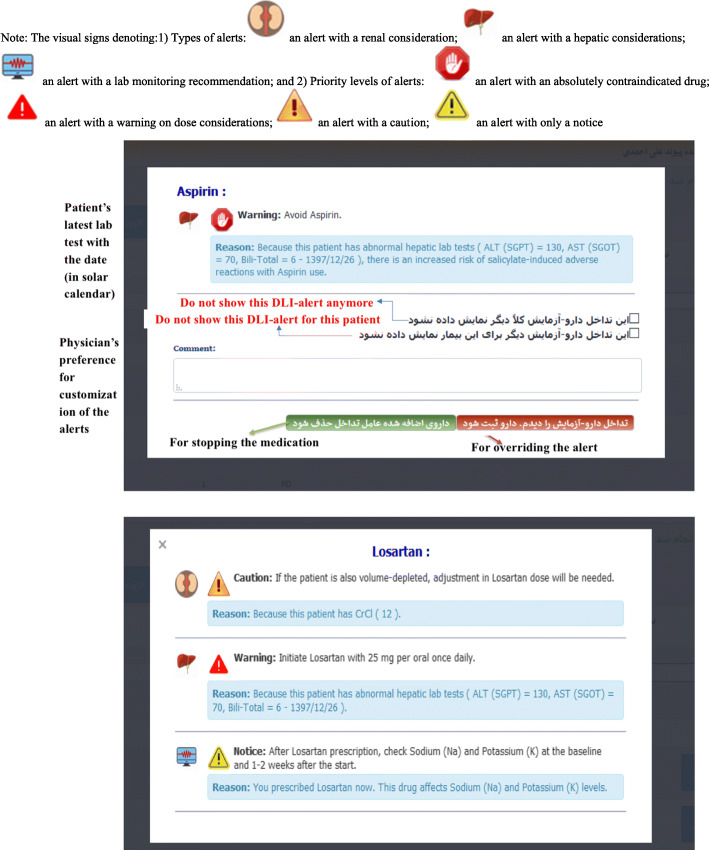
Two screenshots of the DLI-CDSS for a test patient with renal and hepatic impairments

**Fig. 4:**
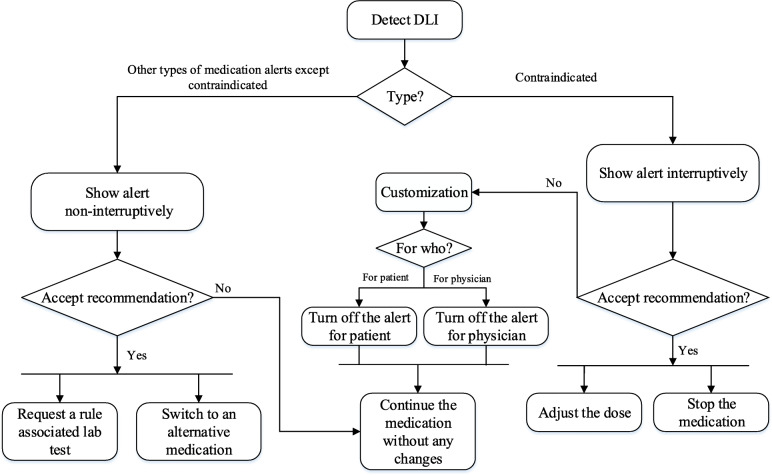
Decision workflow embedded in the DLI-CDSS

Due to poly-pharmacy of kidney recipients, displaying alerts to physicians and interrupting their workflow with each rule would deluge them with a large number of alerts. To prevent this, we considered alerts to be interruptive only when *stopping* a medication or *adjusting* the dose would be necessary, i.e., level 1 severity alert. A pop-up alert was presented to the physician with a message containing the medication name, a recommendation on what to do, a reason accompanying the patient’s latest rule-associated lab value and its date, and a couple of visual clues. Figure 3 provides two screenshots of the system for alerts’ display. The severity levels 2 and 3 appeared on the prescription window without interrupting physician workflow and only in an informational way. In these cases, a small icon with the sign of monitor appeared in front of the medication. To read the alert, a physician could either hover over the medication name and see the alert’s content as a screen tip, or click on it to see the content as a pop-up window. Figure 4 provides the workflow embedded in this CDSS. As a single medication could generate multiple alerts, they were displayed on a single screen.

#### Actions taken in response to alerts

Only the level 1 alerts required a reaction. This reaction could be “override this alert” or “stop medication”. To minimize the impact on an individual physician’s workflow, physicians could decide to keep, revise, or delete a medication in response to an alert. They could also decide to order or not order any rule-associated lab tests (Fig. 4). Althoughthe alerts could be overridden without any further action, they would reappear with every subsequent prescription, including refills. Each trigger of the rules and physicians’ reactions to them were registered in the system for future evaluation of the relevancy of alerts based on the complexities of kidney recipients’ clinical conditions, and to promote the compliance of nephrologists.

### Piloting of the system

System testing was iteratively performed by the research team, by testing the logic embedded in the system against fictitious and the real patients. After the first round of design, the sensitivity and specificity of the designed system were suboptimal (sensitivity of 76% and specificity of 96%) due to several bugs in the system. We fixed the identified bugs and continued testing the system accordingly until no further problems were detected and we achieved 100% in sensitivity and specificity of the system in detecting DLIs of the fictitious and real patients. When the system was ready from a technical point of view, we pilot tested it to detect DLIs in data of 100 real adult patients collected from our clinic. These patients were commonly male (56%) with a mean age of 47.44 years (20 to 73) (Table 2). The system correctly detected 260 DLIs in these 100 patients and displayed 249 monitoring, seven hepatic, four pregnancy, and none renal alerts.

**Table 2:** Characteristics of 100 real patients whose data was used to validate the CDSS system

Characteristics	No. of patients (equals to %)
Male gender	56
Age (year)	
Mean	47.44
Range	20 to 73
Weight (kilogram)	
Mean	72.07
Range	45 to 135
Immunosuppressive medications	
Mycophenolate	74
Cyclosporine	71
Sirolimus	23
Non-immunosuppressive medications	
Diltiazem	51
Calcium	50
Omeprazole	39
Losartan	34
Atorvastatin	27
Lab tests (Mean ± standard deviation)	
Cr	1.57 ± 0.66
AST	19.64 ± 8.16
ALT	21.14 ± 13.24

Abbreviations: *Cr* Creatinine, *AST* Aspartate aminotransferase, *ALT* Alanine aminotransferase

### User satisfaction

Overall, our participants were highly satisfied with the system (4.14 (0.44), mean (SD) in a five-point Likert scale). Regarding the screen factors, the system scored 4.6 (0.24). Users rated the system 4.4 (0.38) in the terminology and system feedback and 3.9 (0.44) in the learning factors aspects. The system scored 3.76 (0.46) regarding the system capabilities.

## Discussion

To our knowledge, this is the first DLI-CDSS to support physicians’ safe medication prescribing for vulnerable transplant recipients based on their lab values. The context and complexities in this field were examined and a system was designed to provide clinicians with a friendly, time-saving, and efficient tool, helping them to consider patients’ demographics, clinical parameters, and lab values in their prescriptions. By using the system, nephrologists can quickly comprehend whether discontinuation, switching to an alternative medication, dose adjustment, or lab monitoring is required based on their patients’ latest lab values. To avoid the alert fatigue, the alert appearance was decided to be interruptive only when medication with a serious risk needed to be discontinued or adjusted. Other alerts are appearing in a non-interruptive mode yet with visual clues on the prescription window to enable easy and intuitive notice. Overall, our clinicians were satisfied with the system, valued its availability, and expressed their interest to use the system in their daily practice. At the time of this report, we have started the implementation of this system for our nephrologists and are planning to evaluate its impact on preventing DLIs in this patient population.

Despite positive effects, various factors may deter CDSS use by clinicians, including their functional features and alert appropriateness [9, 38, 39], which mainly refer to targeting the right provider, on the right issue, in the right place, at the right time, and by the right mode. Among the underlying reasons for non-compliance, lower levels of expected potential harms/benefits for a warning or reminder play an important role [40, 41]. Despite the physicians’ recognition of the value of computerized alerts, they demand individualized alerts for their practices and warn that alerts must not interrupt the workflow or require too many clicks [42]. To address such issues and to comply with the related recommendations (such as [20, 43–47]), we categorized the alert’s significance in three levels of high, medium, and low, of which only the high (level 1) was considered to appear as interruptive pop-ups. This was mainly because we did not want to overwhelm clinicians with many interruptive warnings that most probably would end up in alert fatigue and then be ineffective. Moreover, we selected the most relevant alerts and reminders based on our transplant care context and workflow routines, aiming to increase alert specificity by a context-aware CDSS and to reduce alert overrides as recommended in the literature [48, 49]. Yet, the system was able to fire on average three drug-lab alerts for individual patients. This number is relatively less from what was found in drug labels for individual drugs [43]. To ease the comprehension, we applied screen tips and color codes denoting the priority levels for visual clues that allow physicians to get more information at a glance in a timely manner. In addition, we used only a concise text about the main warnings or reminders and a reason with the patient’s latest rule-relevant lab results. These user interfaces and alert presentation modes were aligned with the extracted user needs/requirements and recommended practice for promoting alert appropriateness aiming to increase clinician compliance [20, 44–47].

Different aspects should be emphasized in the continuous maintenance of CDSSs [50]. The workload pertaining to the management of embedded knowledge within CDSSs becomes even more important when one considers the large number of medications being introduced each year. On the one hand, there are commercial knowledge bases readily accessible for larger organizations with adequate resources that require customizations based on institution and specialty to ensure avoidance of errors in practice [51, 52]. On the other hand, access to CDSS may not be the case for smaller organizations due to costs entailed. Then, they would need to invest in developing in-house small scale knowledge bases, covering their most immediate needs, which would, in turn, require frequent updates with new evidence. It has been recommended that in such cases, updates should be monitored by assigned people regularly [21]. The accuracy of patient records is another aspect of knowledge management: inaccurate or out-of-date records would trigger inaccurate alerts [50]. Therefore, monitoring of the accuracy of patients’ records and addressing problems encountered would lead to clinicians’ reliance on such systems’ advice. The third issue related to CDSS’ maintenance involves evaluating and controlling alert fatigue and override rates [21, 53]. A study in an outpatient setting showed that about half of alerts were overridden from which only half were justifiable [54]. Another study showed that providers continued the same drug at 91.7% of orders despite a warning message probably because they believe the benefits outweigh the potential downsides [16]. In this context, evaluating the appropriateness of alerts based on clinical contexts and workflow becomes especially important [46]. Organizations embarking on CDSS systems need to consider such maintenance efforts in their contexts and secure adequate resources for sustainable CDSS use, in advance.

The medication process is intrinsically multidisciplinary and collaborative [55], in which clinical pharmacists and patients can also play a role besides physicians. However, the evidence about DLI-CDSSs exploiting pharmacists’ inputs in this process is sparse [38]. Currently, our clinical pharmacists are not actively involved in the double-checking of drug interactions; hence, we did not consider them as a user. In studies conducted in outpatient settings, the pharmacists, and not physicians, were those who received electronic alerts on missing laboratory results [11, 56]. Pharmacists had to order tests, remind patients to undergo tests, and review and manage abnormal results, which resulted in significantly improved lab monitoring. In other studies, pharmacists received computerized alerts based on elderly patients’ renal function and communicated the need for dose adjustment to both patients and their physicians [57, 58]. In a population-based randomized clinical trial, the proportion of medication errors was significantly lower in the DLI-CDSS group that alerted pharmacists when compared to the usual care group [59]. To close the loop of medication monitoring, patients themselves are also important. Hence, interventions targeting them alongside health professionals would improve laboratory monitoring and medication safety [42, 60]. Future studies are recommended to target patients, especially those with frequent DLI-CDSSs [60].

In the post kidney transplantation phase, the fertility of women is rapidly restored [61]. However, it accompanies the risk of side effects from immunosuppressive medications and potential renal, maternal (e.g., preeclampsia) and neonatal adverse outcomes [62–64]. Due to the higher risk compared to the general population, pre-pregnancy, antenatal, and postpartum care of renal recipients is highly recommended [65]. Notably, there are instances where prescribing a drug is contraindicated in pregnancy (see for example [66]). Drug–pregnancy alerts represent a category of advanced CDSSs [18]. Significant decreases are shown in physician violations of drug-pregnancy alerts with a CDSS [40]. As one of the main safety features of our CDSS in this patient population, a set of drug-pregnancy alerts were considered to appear with a positive pregnancy test to promote the safety of both mother and fetus.

### Limitations

Althoughto our knowledge, this is the first study to focus on a patient- and context-specific DLI-CDSS in the transplantation domain, it has limitations. To develop a highly context-aware system with a higher specificity, we only included high volume medications used. This limits its overall applicability beyond kidney transplant patients. Nevertheless, it provides a platform that can easily be expanded to include medications of other specialties. Second, our patients were a chronic patient population closely monitored based on available post-transplant clinical practice guidelines (such as [67]). Therefore, we excluded those alerts judged to be redundant to our nephrologists such as a reminder for immunosuppressive level monitoring or Cr test. Third, we relied on estimated CrCl calculated using serum Cr as a cheap and routine parameter for kidney function; but, we are aware that this is not an exact measure of glomerular filtration rate. Fourth, the impact of drugs on the diagnostic interpretation of laboratory tests was out of the scope of our study (see for example [68]). The last but not the least, the result of our satisfaction study suggests that the system was generally well received by clinicians. Despite this, the impact of our efforts to address usefulness, usability, and alert appropriateness, alert compliance and patient outcomes need to be supported by future well-performed studies.

## Conclusion

A multidisciplinary team developed a DLI-CDSS with the patient- and context-specific warnings and reminders on dosing recommendations and conflicts with patients’ lab-based safety considerations. We hope that with such an approach, DLIs can be more promptly recognized and more reliably addressed. However, we were aware that such systems would not be embraced by physicians if they are overwhelmed by a blizzard of poorly validated warnings. To account for this, we used the high priority DLI rules validated by clinicians. It is plausible that being vigilant to the side effects of medications based on lab values would promote patient safety. This should be documented by future studies.

## Supplementary information

**Additional file 1.** Formulas for CrCl and LBW.

**Additional file 2.** Complete set of 27 medications.

**Additional file 3.** Sample diagrams for individual drugs.

**Additional file 4.** Sample diagrams for individual drugs.

**Additional file 5.** Sample diagrams for individual drugs.

**Additional file 6.** Sample diagrams for individual drugs.

## Data Availability

The datasets supporting the conclusions of this article are included within the article (and its additional files).

## Abbreviations

DLI: Drug-Laboratory Interaction
CDSS: Clinical Decision Support System
pADE: Preventable Adverse Drug Events
UMSU: Urmia Medical Science University
RTMS: Renal Transplant Management System
CPOE: Computerized Provider Order Entry
CrCl: Creatinine Clearance
LBM: Lean Body Mass
UML: Unified Modeling Language
LFT: Liver Function Test
Cr: Creatinine
AST: Aspartate aminotransferase
ALT: Alanine aminotransferase
