# Supplementary material for: Translation of evidence into kidney transplant clinical practice: managing drug-lab interactions by a context-aware clinical decision support system
Source: BMC Med Inform Decis Mak. 2020 Aug 20;20:196. doi: 10.1186/s12911-020-01196-w (PMC7439664; doi:10.1186/s12911-020-01196-w)
Supplement: Supplementary file 1 — Additional file 1. Formulas for CrCl and LBW. [file 12911_2020_1196_MOESM1_ESM.docx]

**Formulas embedded in the CDSS for automatic calculation**

- **The Cockcroft and Gault formula** ([1](#_ENREF_1))

$$CrCl \left( \frac{ml}{\min} \right)=k\times\frac{\left[ \left( 140-age \right)\times weight \left( kg \right) \right]}{SCr\left( \frac{mg}{dl} \right)\times72}$$

For female gender, k = 0.85 was used.

- **The Hume formula for the lean body weight/mass (LBM)** in our diagrams for digoxin was also automatically calculated by the system according to the Hume formula as the following ([2](#_ENREF_2)):

For males:

$$eLBM = 0.32810W + 0.33929H - 29.5336$$

For females:

$$eLBM = 0.29569W + 0.41813H - 43.2933$$

In which, the W is the body weight in kilogram and the H is the height in centimeter.

**References:**

1. Cockcroft D, Gault M. Prediction of creatinine clearance from serum creatinine. . Nephron. 1976;16:31-41.

2. Hume R. Prediction of lean body mass from height and weight. Journal of Clinical Pathology. 1966;19(4):389-91.
