# Supplementary material for: Translation of evidence into kidney transplant clinical practice: managing drug-lab interactions by a context-aware clinical decision support system
Source: BMC Med Inform Decis Mak. 2020 Aug 20;20:196. doi: 10.1186/s12911-020-01196-w (PMC7439664; doi:10.1186/s12911-020-01196-w)
Supplement: Supplementary file 2 — Additional file 2. Complete set of 27 medications. [file 12911_2020_1196_MOESM2_ESM.docx]

Additional file 2

Complete set of drug-lab interaction guidelines for the top 27 high-volume medications in our study

|  | Medication * | ATC category and name ^α^ | Dose adjustment in renal impairment | Dose adjustment in hepatic impairment | Pregnancy considerations | Lab monitoring considerations |
| --- | --- | --- | --- | --- | --- | --- |
| 1 | Aciclovir | J05: ANTIVIRALS FOR SYSTEMIC USE | - 10 ≤ CrCl < 24 ml/min/1.73 m²: extend dosage interval to every 8h, if patient receives 800 mg per oral 5 times per day. - CrCl < 10 ml/min/1.73 m²: reduce dose to 800 mg per oral every 12 hour, if patient receives 800 mg per oral 5 times per day. | No adjustment is required | ---- | ---- |
| 2 | Allopurinol | M04: ANTIGOUT PREPARATIONS | - 10 ≤ CrCl < 20 ml/min/1.73m²: prescribe 200 mg/day per oral - 3 ≤ CrCl < 10 ml/min/1.73m²: prescribe 100 mg/day per oral - CrCl < 3 ml/min/1.73m²: prescribe 100 mg per oral every 24 hour or longer; or 100 mg per oral every third day. | Dosage adjustment may be necessary; No specific recommendations available | ---- | - Monitor Uric acid level: - If normal, check it every 6 months. - If abnormal, change Allopurinol dose accordingly |
| 3 | Amlodipine | C08: CALCIUM CHANNEL BLOCKERS | No adjustment is required | - In hepatic impairment (ALT > 40 U/ml OR AST > 40 U/L OR Bili-total > 1.5 mg/dl): initiate adult dose at 2.5 mg per oral once daily for hypertension or 5 mg per oral once daily for angina | --- | --- |
| 4 | Aspirin (acetylsalicylic acid) | B01: ANTITHROMBOTIC AGENTS | - CrCl < 10 ml/min/1.73 m²: avoid | - In severe hepatic disease (AST > 120 U/L OR ALT > 120 U/ml OR Bili-total > 1.5 mg/d): avoid | --- | --- |
| 5 | Atenolol | C07: BETA BLOCKING AGENTS | - 15 ≤ CrCl < 35 ml/min/1.73 m²: do not exceed 50 mg/day. - CrCl < 15 ml/min/1.73 m²: do not exceed 25 mg/day. | No adjustment is required | Discontinue | --- |
| 6 | Atorvastatin | C10: LIPID MODIFYING AGENTS | No adjustment is required | - In active liver disease (ALT > 200 U/mL OR AST > 200 U/L OR Bili-total > 1.5) OR unexplained elevated transaminase for 3 weeks or 3 months (ALT > 40 U/mL OR AST > 40 U/L): discontinue | Discontinue | --- |
| 7 | Azathioprine | L04: IMMUNOSUPPRESSANTS | - If renal impairment or oliguria exists, then dosage should be modified depending on clinical response and degree of renal impairment. No quantitative recommendations are available. | Specific guidelines for dosage adjustments in hepatic impairment are not available | Discontinue | --- |
| 8 | Calcium | A12: MINERAL SUPPLEMENTS | No adjustment is required | No adjustment is required | --- | - Monitor serum calcium, phosphate and iPTH |
| 9 | Captopril | C09: AGENTS ACTING ON THE RENIN-ANGIOTENSIN SYSTEM | - 10 ≤ CrCl < 50 ml/min/1.73 m²: reduce the recommended dose by 25%. - CrCl < 10 ml/min/1.73 m²: reduce the recommended dose by 50%. | No adjustment is required | Discontinue | - Monitor Na and K at the baseline and 1-2 weeks after the start. |
| 10 | Ciprofloxacin | J01: ANTIBACTERIALS FOR SYSTEMIC USE | - 10 ≤ CrCl < 50 ml/min/1.73m²: administer 50 to 75% of the regular dose. - CrCl < 10 ml/min/1.73m: administer 50% of the regular dose. | No adjustment is required | --- | --- |
| 11 | Cotrimoxazol (Sulfamethoxazole and Trimethoprim) | J01: ANTIBACTERIALS FOR SYSTEMIC USE | - 15 ≤ CrCl < 30 ml/min/1.73 m²: reduce dose by 50% - CrCl < 15 ml/min/1.73 m²: administration is not recommended. | Dosage adjustments may be necessary in patients with hepatic impairment, however, specific dosage adjustment guidelines are not available | --- | - If renal impairment exists, check and monitor serum K. |
| 12 | Cyclosporine | L04: IMMUNOSUPPRESSANTS | No adjustment is required | - In hepatic impairment (ALT > 40 U/mL OR AST > 40 U/L OR Bili-total > 1.5 mg/dl): monitor Cyclosporine blood concentration level. May require dose reduction based on concentration. | --- | - Monitor Uric acid, K, and Mg levels every 2 weeks in the first 3 months then monthly |
| 13 | Digoxin | C01: CARDIAC THERAPY | - CrCl 90 to 99 ml/min/1.73m²:   - LBW 40 to 49 kg: Administer Digoxin 187.5 mcg per oral once daily.   - LBW 50 to 69 kg: Administer Digoxin 250 mcg per oral once daily.   - LBW 70 to79 kg: Administer Digoxin 312.5 mcg per oral once daily.   - LBW 80 to 89 kg: Administer Digoxin 375 mcg per oral once daily.   - LBW 90 kg or more: Administer Digoxin 437.5 mcg per oral once daily. - CrCl 80 to 89 ml/min/1.73m²:   - LBW 40 to 59 kg: Administer Digoxin 187.5 mcg per oral once daily.   - LBW 60 to 69 kg: Administer Digoxin 250 mcg per oral once daily   - LBW 70 to 89 kg: Administer Digoxin 312.5 mcg per oral once daily.   - LBW 90 to 99 kg: Administer Digoxin 375 mcg per oral once daily.   - LBW 100 kg or more: Administer Digoxin 437.5 mcg per oral once daily. - CrCl 70 to 79 ml/min/1.73m²:   - LBW 40 to 59 kg: Administer Digoxin 187.5 mcg per oral once daily.   - LBW 60 to 79 kg: Administer Digoxin 250 mcg per oral once daily.   - LBW 80 to 89 kg: Administer Digoxin 312.5 mcg per oral once daily.   - LBW 90 kg or more: Administer Digoxin 375 mcg per oral once daily. - CrCl 60 to 69 ml/min/1.73m²:   - LBW 40 to 49 kg: Administer Digoxin 125 mcg per oral once daily   - LBW 50 to 59 kg: Administer Digoxin 187.5 mcg per oral once daily.   - LBW 60 to 79 kg: Administer Digoxin 250 mcg per oral once daily.   - LBW 80 to 99 kg: Administer Digoxin 312.5 mcg per oral once daily.   - LBW 100 kg or more: Administer Digoxin 375 mcg per oral once daily. - CrCl 40 to 59 ml/min/1.73m²:   - LBW 40 to 49 kg: Administer Digoxin 125 mcg per oral once daily.   - LBW 50 to 69 kg: Administer Digoxin 187.5 mcg per oral once daily.   - LBW 70 to 89 kg: Administer Digoxin 250 mcg per oral once daily.   - LBW 90 kg or more: Administer Digoxin 312.5 mcg per oral once daily. - CrCl 30 to 39 ml/min/1.73m²:   - LBW 40 to 59 kg: Administer Digoxin 125 mcg per oral once daily.   - LBW 60 to 79 kg: Administer Digoxin 187.5 mcg per oral once daily.   - LBW 80 to 99 kg: Administer Digoxin 250 mcg per oral once daily.   - LBW 100 kg or more: Administer Digoxin 312.5 mcg per oral once daily. - CrCl 20 to 29 ml/min/1.73m²:   - LBW 40 to 69 kg: Administer Digoxin 125 mcg per oral once daily.   - LBW 70 to 89 kg: Administer Digoxin 187.5 mcg per oral once daily.   - LBW 90 kg or more: Administer Digoxin 250 mcg per oral once daily. - CrCl < 20 ml/min/1.73m²:   - LBW 40 to 49 kg: Administer Digoxin 62.5 mcg per oral once daily.   - LBW 50 to 69 kg: Administer Digoxin 125 mcg per oral once daily.   - LBW 70 to 99 kg: Administer Digoxin 187.5 mcg per oral once daily.   - LBW 100 kg or more: Administer Digoxin 250 mcg per oral once daily | No adjustment is required | --- | --- |
| 14 | Diltiazem | C08: CALCIUM CHANNEL BLOCKERS | No adjustment is required | - In hepatic impairment (ALT > 40 U/mL OR AST > 40 U/L OR Bili-total > 1.5 mg/dl): Reduce dose by 50% and adjust the dose based on clinical responses. | --- | --- |
| 15 | Enalapril | C09: AGENTS ACTING ON THE RENIN-ANGIOTENSIN SYSTEM | - CrCl ≤ 30 ml /min/1.73m²: Reduce initial dose to 2.5 mg per oral once daily. | No adjustment is required | Discontinue | - Monitor Na and K at the baseline and 1-2 weeks after the start. |
| 16 | Ferfolic | B03: ANTIANEMIC PREPARATIONS | No adjustment is required | Specific guidelines for dosage adjustments in hepatic impairment are not available | --- | - Monitor the followings every 3-6 months:   - Serum Ferritin   - Total Iron-Binding Capacity   - Serum Iron Concentration |
| 17 | Furosemide | C03: DIURETICS | No adjustment is required | - In hepatic impairment (ALT > 40 U/mL OR AST > 40 U/L OR Bili-total > 1.5 mg/dl): use with caution | --- | - Monitor serum electrolytes regularly. |
| 18 | Gemfibrozil | C10: LIPID MODIFYING AGENTS | - 10 ≤ CrCl < 50 ml/min/1.73 m²: consider an alternative therapy. Use with caution if Gemfibrozil is necessary. - CrCl < 10 ml/min/1.73 m²: Avoid | - In hepatic impairment (ALT > 40 U/mL OR AST > 40 U/L OR Bili-total > 1.5 mg/dl) and cirrhotic liver disease: discontinue. This drug is contraindicated in cirrhotic liver disease. | Discontinue | - Monitor serum cholesterol every 6 months |
| 19 | Hydrochlorothiazide | C03: DIURETICS | - CrCl < 30 ml/min/1.73 m²: do not use. | - In hepatic impairment (ALT > 40 U/mL OR AST > 40 U/L OR Bili-total > 1.5 mg/dl): use with caution, since minor alteration of fluid and electrolyte balance may precipitate hepatic coma. | --- | - Monitor K, Na and Cl |
| 20 | Isoniazid | J04: ANTIMYCOBACTERIALS | - CrCl < 10 ml/min//1.73 m²: consider a reduction of dose to 150-200 mg/day (up to a 50% dosage reduction). | - In patients with a history of hepatic disease (ALT > 40 U/mL OR AST > 40 U/L OR Bili-total > 1.5 mg/dl): use only after symptoms and laboratory abnormalities of mild and moderate hepatic impairment have cleared. - In acute hepatic disease (ALT > 120 U/mL OR AST > 120 U/L OR Bili-total ≥ 3 mg/dl): avoid. | ----- | ----- |
| 21 | Losartan | C09: AGENTS ACTING ON THE RENIN-ANGIOTENSIN SYSTEM | - CrCl < 30 ml /min/1.73m²: If the patient is also volume-depleted, dose adjustment will be needed. | - In hepatic impairment (ALT > 40 U/mL OR AST > 40 U/L OR Bili-total > 1.5 mg/d): initiate with 25 mg per oral once daily. | Discontinue | - Monitor Na and K at the baseline and 1-2 weeks after the start |
| 22 | Metoprolol | C07: BETA BLOCKING AGENTS | No adjustment is required | - In hepatic impairment (ALT > 40 U/mL OR AST > 40 U/L OR Bili-total > 1.5 mg/d): initiate at 25 mg per oral once daily and titrate slowly according to clinical responses. | --- | --- |
| 23 | Mycophenolat (mycophenolic acid) | L04: IMMUNOSUPPRESSANTS | - CrCl < 25 ml/min/1.73 m²: do not exceed 1 gr per oral twice daily. | No adjustment is required | Discontinue | - Monitor pregnancy test |
| 24 | Omeprazole | A02: DRUGS FOR ACID RELATED DISORDERS | No adjustment is required | - In severe hepatic disease (AST > 120 U/L OR ALT > 120 U/ml OR Bili-total > 3 mg/d) and cirrhotic liver disease: reduce Omeprazole dose to 10 mg once daily receiving for long-term therapy. | ----- | - Monitor Mg and Vit B12 levels periodically |
| 25 | Ranitidine | A02: DRUGS FOR ACID RELATED DISORDERS | - CrCl < 50 ml/min/1.73m²: reduce the recommended dose of Ranitidine by 50% (or extend dosing interval). | No adjustment is required | --- | --- |
| 26 | Sirolimus | L04: IMMUNOSUPPRESSANTS | No adjustment is required | - In mild and moderate hepatic impairment (ALT > 40 U/mL OR AST > 40 U/l OR Bili-total > 1.5 mg/dl) OR (Child-Pugh class A OR B): reduce the dose by 1/3 of maintenance dose. - In severe hepatic impairment (Child-Pugh class C) or (ALT > 120 U/mL OR AST > 120 U/l OR Bili-total ≥ 3 mg/dl): reduce the dose by 1/2 of maintenance dose. | ---- | ------ |
| 27 | Tacrolimus | L04: IMMUNOSUPPRESSANTS | - In renal impairment: reduce the dose and check Cr level after one week. If patient has persistent serum creatinine concentration elevations despite a dose reduction, switch Tacrolimus to a different immunosuppressive therapy. | - In patients with severe hepatic impairment (Child-Pugh class C) or (ALT > 120 U/mL OR AST > 120 U/l OR Bili-total ≥ 3 mg/dl): reduce the dose due to reduced Tacrolimus clearance and prolonged half-life. | --- | - Monitor Mg, P, and K |

* Medications are ordered alphabetically

^α^ Drug categories are according to the WHO’s ATC standard

Abbreviations: Anatomical Therapeutic Chemical-code (ATC-code); Creatinine clearance (CrCl), Aspartate aminotransferase (AST) and Alanine aminotransferase (ALT), Bilirubin (Bili), Magnesium (Mg); Phosphorus (P); Potassium (K); intact parathyroid hormone (iPTH), Lean Body Weight (LBW)
