# Supplementary figures and images for: Translation of evidence into kidney transplant clinical practice: managing drug-lab interactions by a context-aware clinical decision support system
Source: BMC Med Inform Decis Mak. 2020 Aug 20;20:196. doi: 10.1186/s12911-020-01196-w (PMC7439664; doi:10.1186/s12911-020-01196-w)

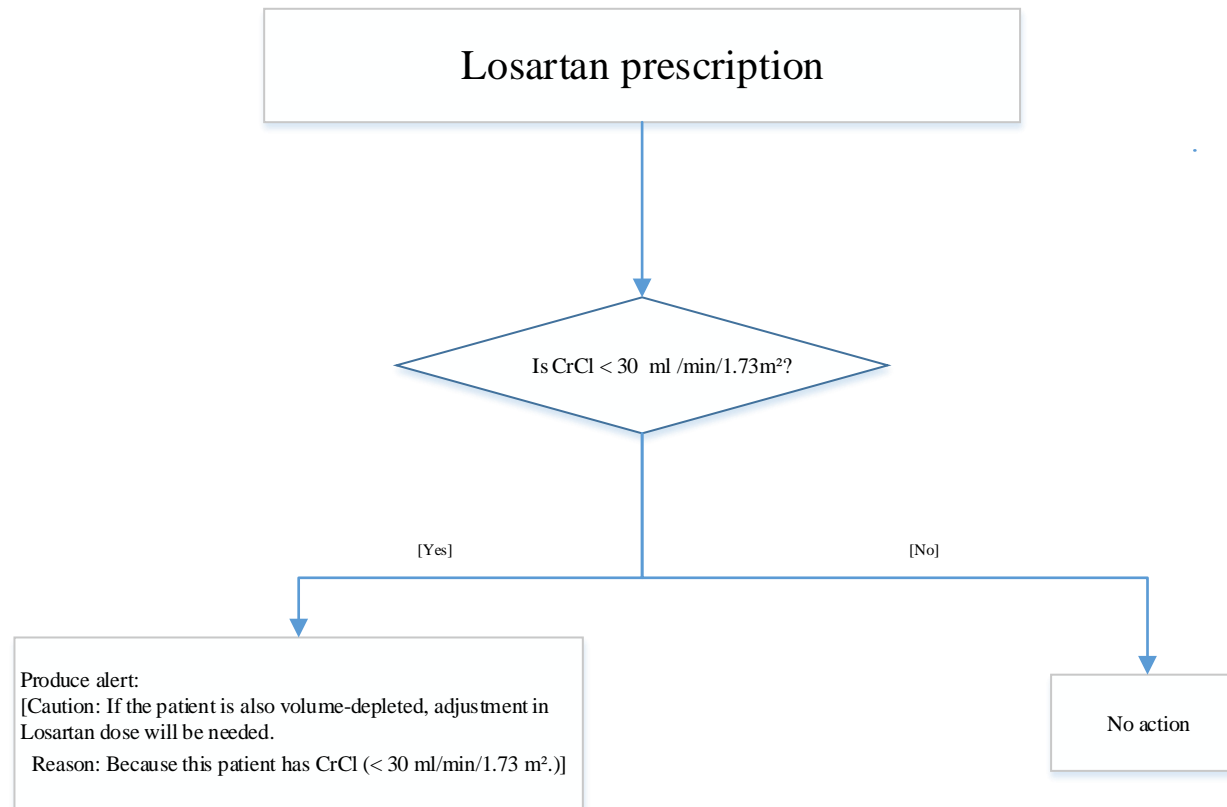

Supplement: Supplementary file 3 — Additional file 3. Sample diagrams for individual drugs. [file 12911_2020_1196_MOESM3_ESM.pdf]

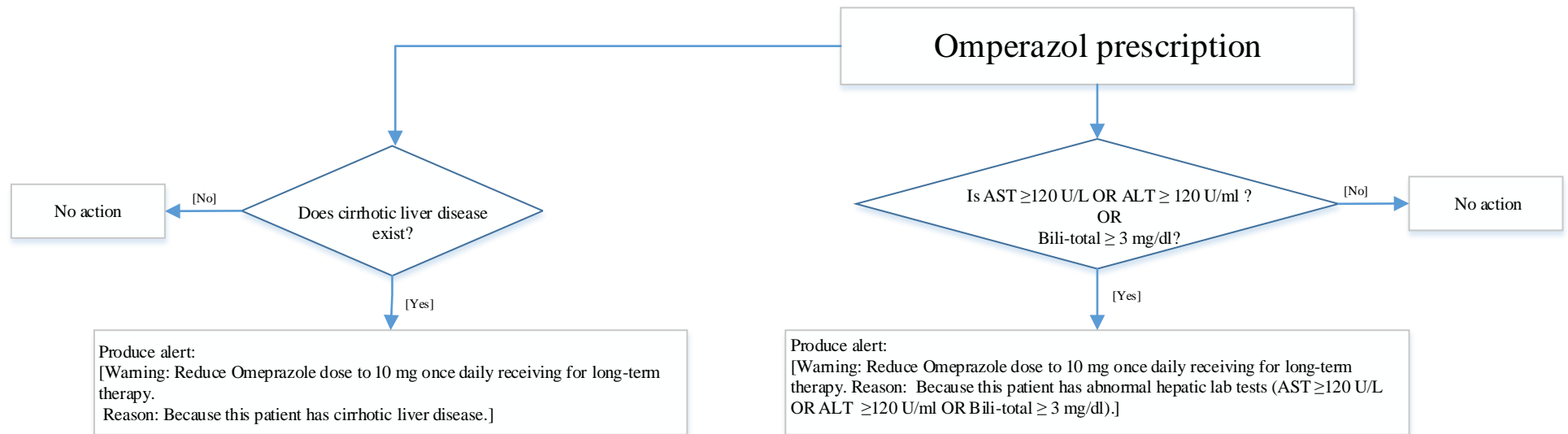

Supplement: Supplementary file 4 — Additional file 4. Sample diagrams for individual drugs. [file 12911_2020_1196_MOESM4_ESM.pdf]

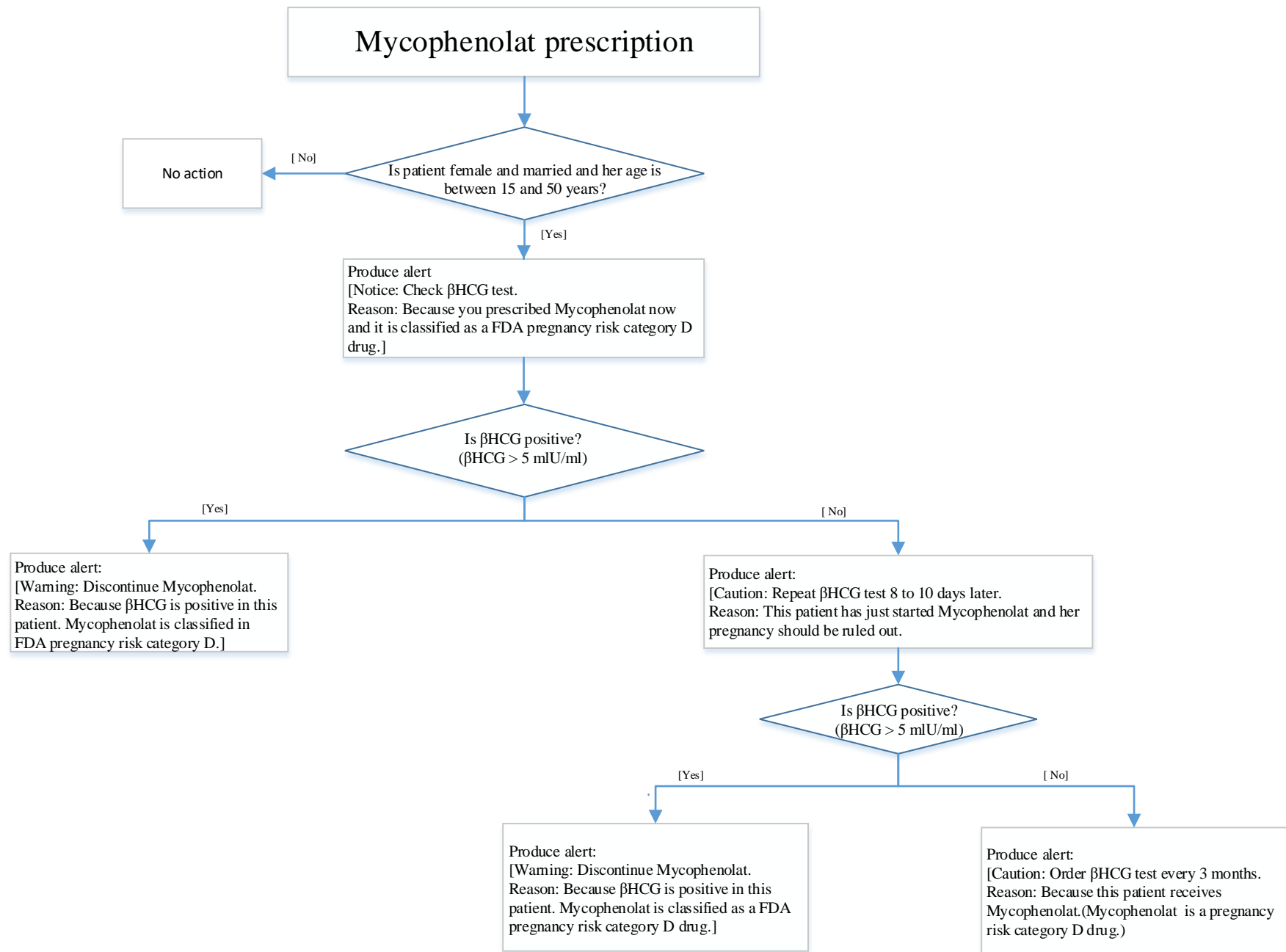

Supplement: Supplementary file 5 — Additional file 5. Sample diagrams for individual drugs. [file 12911_2020_1196_MOESM5_ESM.pdf]

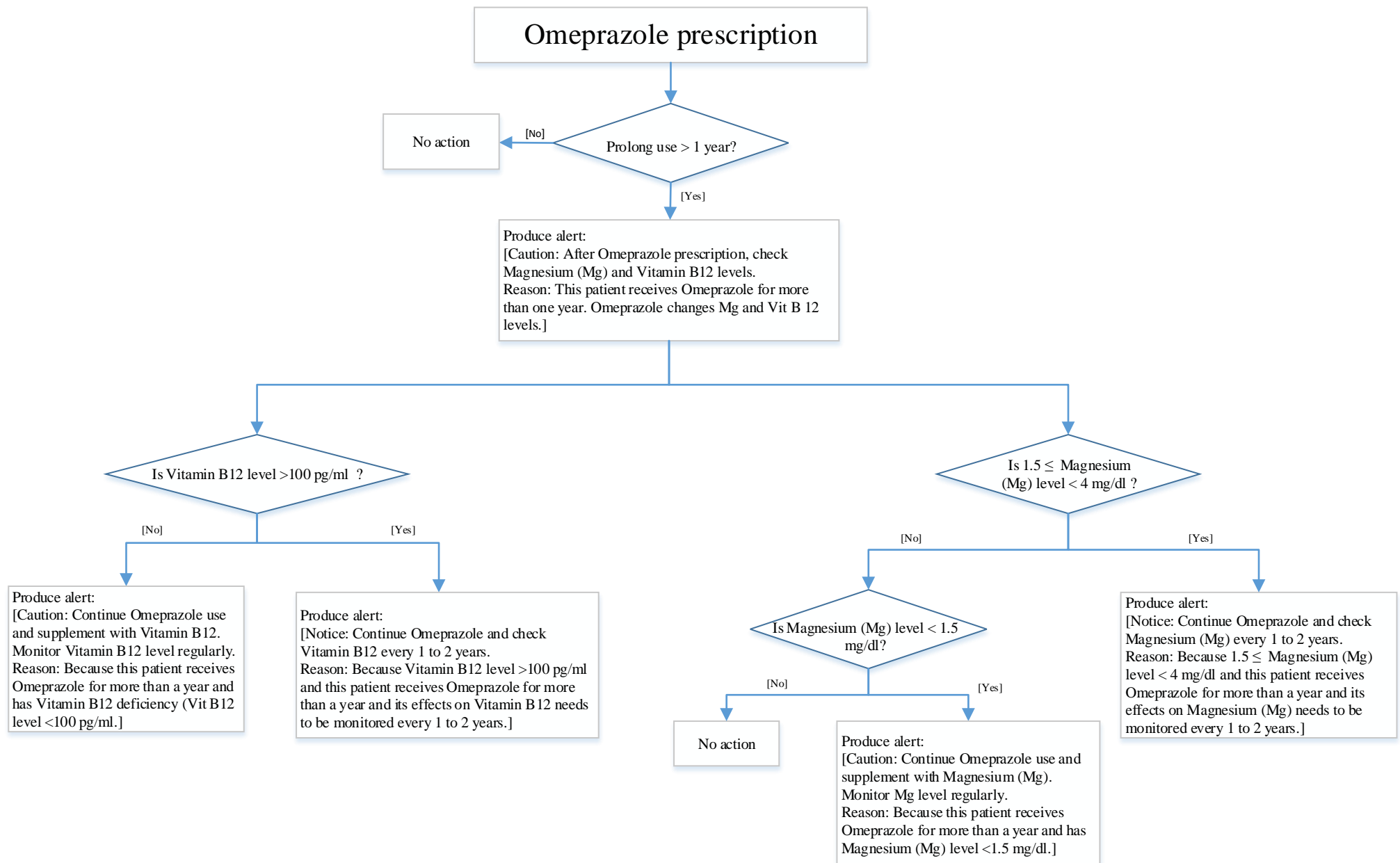

Supplement: Supplementary file 6 — Additional file 6. Sample diagrams for individual drugs. [file 12911_2020_1196_MOESM6_ESM.pdf]
